# Supplementary material for: The interactome of intact mitochondria by cross-linking mass spectrometry provides evidence for coexisting respiratory supercomplexes
Source: Mol Cell Proteomics. 2017 Dec 8;17(2):216–32. doi: 10.1074/mcp.RA117.000470 (PMC5795388; doi:10.1074/mcp.RA117.000470)
Supplement: Supplemental Data [file supp_RA117.000470_133922_0_supp_23978_fzffwf.zip › spectra_annotation/mito_spectra_annotation/325-1-11-1-12-1.pdf]

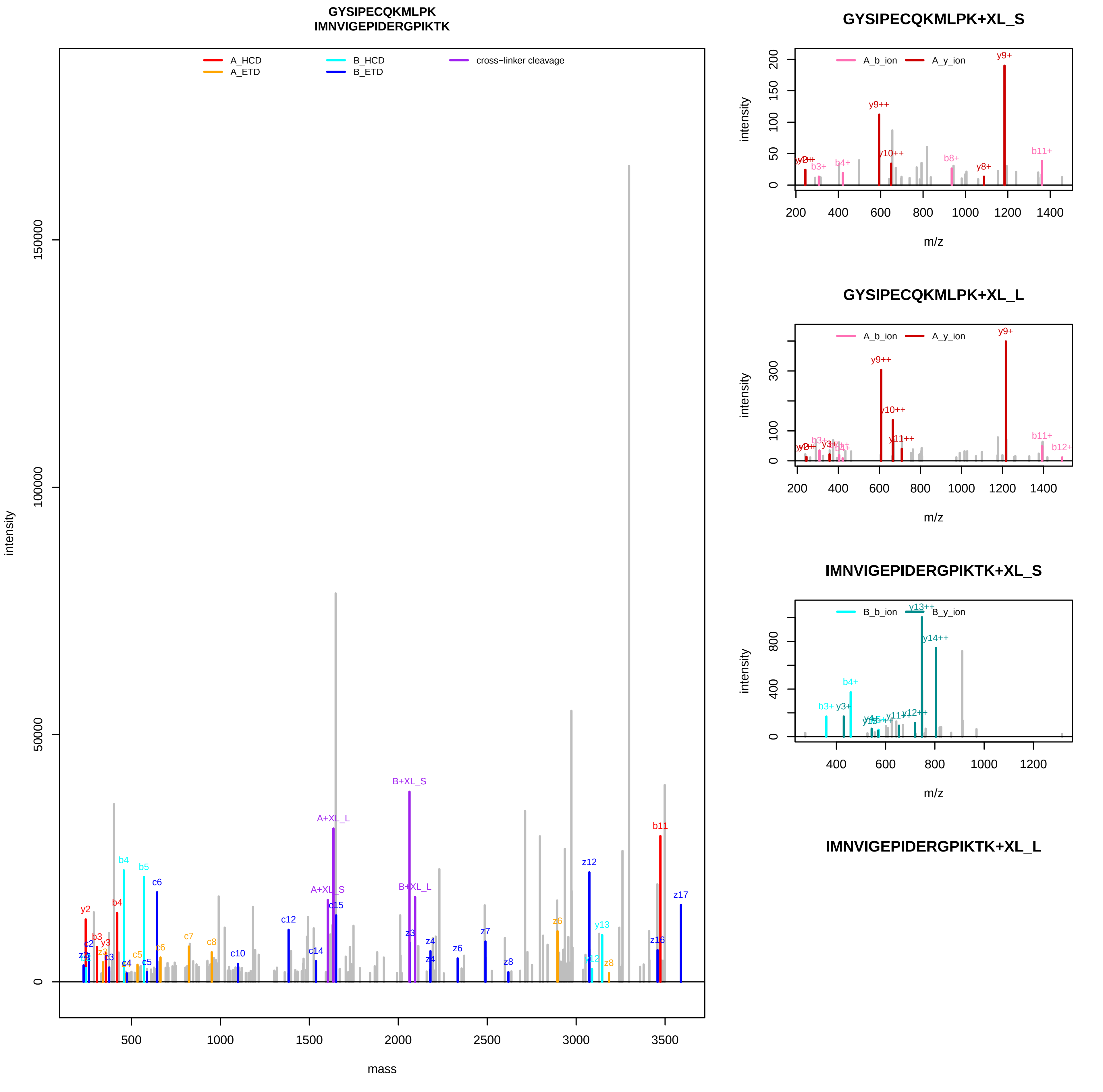
GYSIPECQKMLPK
IMNVIGEPIDERGPIKTK
A_HCD
A_ETD
B_HCD
B_ETD
cross−linker cleavage
intensity
mass
0
50000
100000
150000
500
1000
1500
2000
2500
3000
3500
A+XL_L
A+XL_S
B+XL_S
B+XL_L
b11
z12
z17
c15
c12
c14
c10
c6
b4
b5
y2
y13
z6
z7
z8
GYSIPECQKMLPK+XL_S
A_b_ion
A_y_ion
y9+
y9++
y10++
b11+
b8+
y8+
b4+
b3+
intensity
m/z
GYSIPECQKMLPK+XL_L
A_b_ion
A_y_ion
y9+
y9++
y10++
y11++
b11+
b12+
intensity
m/z
IMNVIGEPIDERGPIKTK+XL_S
B_b_ion
B_y_ion
y13++
y14++
b4+
b3+
y3+
y12++
intensity
m/z
IMNVIGEPIDERGPIKTK+XL_L
